# Aflibercept versus ranibizumab for treating persistent diabetic macular oedema

**DOI:** 10.1007/s10792-015-0081-7

**Published:** 2015-05-20

**Authors:** Kristof ROA Vandekerckhove

**Affiliations:** Vista Alpina eye center, Bahnhofplatz 1A, 3930 Visp, Switzerland

**Keywords:** DME, Ranibizumab, Aflibercept, PlGF, VEGF, Intravitreal

## Abstract

The purpose of this study was to compare the efficacy of intravitreal aflibercept versus ranibizumab for treating therapy-resistant diabetic macular oedema (DME). A 69-year-old man presented with persistent bilateral DME despite previous ranibizumab treatment. Bilateral study treatment comprised one cycle of three monthly ranibizumab injections (0.5 mg), followed by one cycle of three aflibercept injections (2.0 mg), a second ranibizumab cycle and a second aflibercept cycle. Baseline visual acuity (ETDRS score) was 60 letters for the right eye and 65 letters for the left eye. Baseline central foveal thickness (CFT) was 305 μm for the right eye and 453 μm for the left eye. Substantially improved outcomes were observed during the first aflibercept cycle. CFT was reduced by 150 μm (mean) in both the eyes and decreased below the lowest level achieved during the previous 2.5-year ranibizumab treatment. Visual acuity was improved by 17.5 letters (mean) in both the eyes. Reintroduction of ranibizumab immediately worsened the status of both eyes back to the baseline level. During the final aflibercept cycle, visual acuity and CFT improved to the same levels achieved during the first aflibercept cycle. In this case study, we prospectively switched the treatment three times and observed a dramatic and consistent treatment advantage for aflibercept.

**Electronic supplementary material:**

The online version of this article (doi:10.1007/s10792-015-0081-7) contains supplementary material, which is available to authorized users.

## Introduction

Both ranibizumab and aflibercept are indicated for treating DME (Food and Drug Administration approval in 2012 and 2014, respectively) based on the results of phase III data of RISE/RIDE (ranibizumab) and VISTA/VIVID (aflibercept) studies [1, 2].

However, no comparative data are available on the potential differences in the efficacy of ranibizumab and aflibercept for treating persistent or recurrent DME. Given the complex aetiopathogenesis of DME and given the differences in mode of action between the two drugs, clinical efficacy might be different. We report here the results of a case study of persistent bilateral DME, in which intravitreal treatment has been switched back and forth between ranibizumab and aflibercept several times. To the best of our knowledge, this is the first case study to analyse the effect of switching between the two drugs for treating DME.

## Materials and methods

### Patient

This study included a 69-year-old man with type I diabetes who was diagnosed with bilateral DME in 2009. Since February 2011, the patient received intravitreal anti-VEGF treatment (ranibizumab) for both the eyes. Despite 2.5 years of near-monthly ranibizumab treatment (21 injections in both the eyes; average interval, 38 days), intraretinal and subretinal fluid persisted. The patient entered the study in May 2013 (baseline). Baseline values were determined after a one-month washout period from the prestudy treatment (Online Resource 1). The patient did not show any signs of other ophthalmological pathologies besides nonproliferative diabetic retinopathy and DME (Online Resource 2).

### Study design and protocol

**Fig. 1:**
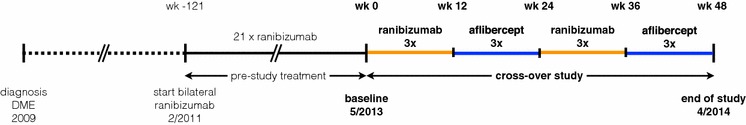
Flowchart describing the study design (one patient, both the eyes). Study injections and examinations were performed at 4-week intervals (±2 days). *wk* week

This prospective case study compared the efficacy of aflibercept with that of ranibizumab for treating refractory DME. This study followed a double-crossover design in which the treatment was switched three times (Fig. 1). Bilateral study treatment included one cycle of three ranibizumab injections (0.5 mg), followed by one cycle of three aflibercept injections (2.0 mg), a second cycle of three ranibizumab injections and a second cycle of three aflibercept injections at 4-week intervals (±2 days).

Primary end-point was the change in mean central foveal thickness (CFT) from baseline, and secondary end-point was the change in mean best-corrected visual acuity (BCVA) from baseline.

CFT was calculated from a 15-line Optopol HR Copernicus (OPTOPOL Technology Sp., Zawiercie, Poland) radial scan with 2812 A-scans per line. Inbuilt Optopol software with image recognition was used to measure CFT. CFT was defined as the distance between the internal limiting membrane and Bruch’s membrane. Anatomical boundaries and CFT values were reviewed by two imaging graders (K.V. and A.E.) to ensure that the automated algorithms accurately identified the foveal location. The central foveal location was manually redefined for only one datapoint.

BCVA was measured using the Snellen chart at 4 m and was converted from the Snellen notation to the ETDRS letter score [3].

### Injection procedure

All the injections were given according to a standardised procedure by a single surgeon (K.V.) in an operating room.

## Results

**Fig. 2:**
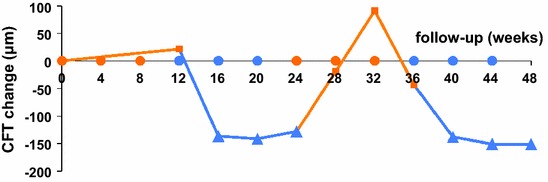
Graph showing changes in mean CFT from baseline over 48 weeks, during treatment with ranibizumab or aflibercept. The mean for both the eyes is shown. Note the consistent response during the ranibizumab and aflibercept treatment cycles. *Orange squares* response 4 weeks after ranibizumab, *blue triangles* response 4 weeks after aflibercept, *orange dots* ranibizumab injections, *blue dots* aflibercept injections, *CFT* Central Foveal Thickness

**Fig. 3:**
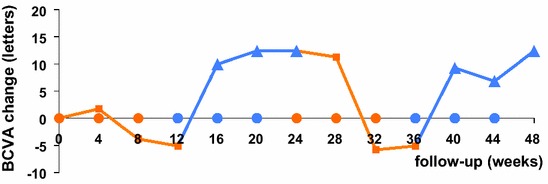
Graph showing changes in mean BCVA (ETDRS letters) from baseline over 48 weeks, during treatment with ranibizumab or aflibercept. The mean for both the eyes is shown. Note the consistent response during the ranibizumab and aflibercept treatment cycles. *Orange squares* response 4 weeks after ranibizumab, *blue triangles* response 4 weeks after aflibercept, *orange dots* ranibizumab injections, *blue dots* aflibercept injections, *BCVA* best-corrected visual acuity, *ETDRS* Early Treatment of Diabetic Retinopathy Study

Unsurprisingly, after 2.5 years of ranibizumab treatment, the first cycle of ranibizumab injections did not have a significant impact on visual acuity or on macular oedema. In contrast, the very first aflibercept injection substantially improved the anatomical and functional outcomes. Macular oedema reduced to below the lowest level achieved during the previous 2.5 years of ranibizumab treatment. Reintroduction of ranibizumab immediately worsened the anatomical and functional status of the patient. BCVA and CFT values reached pre-aflibercept levels by the end of the second ranibizumab cycle. However, reintroduction of aflibercept (second aflibercept cycle) improved the functional and anatomical outcomes to a similar extent as that observed with the first aflibercept cycle. Throughout the study, the evolution of the anatomical and functional outcomes for the right eye closely followed those observed for the left eye (Table 1). These outcomes are graphically presented as the mean of both the eyes (Figs. 2, 3).

**Table 1:** Treatment response at the end of each treatment cycle

	BCVA (letters)		CFT (μm)	
	Absolute value	Change during the cycle	Absolute value	Change during the cycle
Baseline				
RE	60.1		305	
LE	65.1		453	
Both (mean)	62.7		379	
Ranibizumab cycle 1				
RE	50.1	−10.0	301	−4
LE	65.1	0	500	+47
Both (mean)	57.6	−5.1	401	+22
Aflibercept cycle 1				
RE	65.1	+15.0	233	−68
LE	85.0	+19.9	269	−231
Both (mean)	75.1	+17.5	251	−150
Ranibizumab cycle 2				
RE	50.1	−15.0	287	+54
LE	65.1	−19.9	384	+115
Both (mean)	57.6	−17.5	336	+85
Aflibercept cycle 2				
RE	65.1	+15.0	188	−99
LE	85.0	+19.9	268	−116
Both (mean)	75.1	+17.5	228	−108

Baseline = 4 weeks after washout from the prestudy ranibizumab treatment

Treatment cycle = 3 intravitreal injections at 4 week intervals

Treatment response = changes in BCVA and CFT values measured at 4 weeks after the last intravitreal injections of the treatment cycle and compared with the values at the start of each cycle

*BCVA* Best-corrected visual acuity

*EDTRS* Early treatment of diabetic retinopathy study

*CFT* Central foveal thickness

**Fig. 4:**
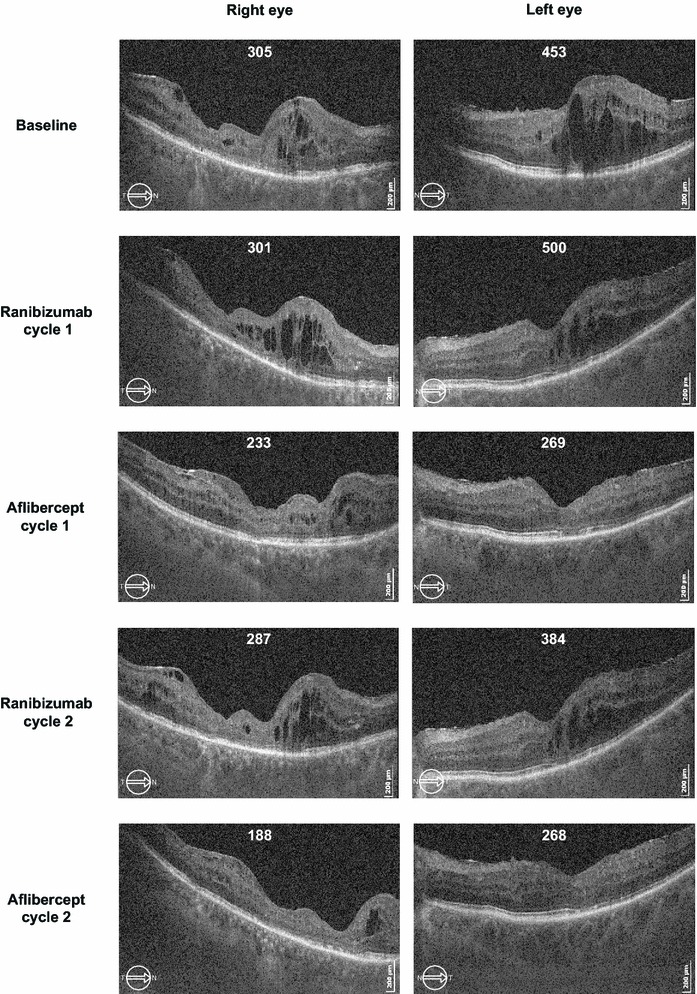
Successive ocular coherence tomography (OCT) of horizontal sections (7.0 mm) from the right and left eyes shows the evolution of subretinal and intraretinal fluid. Central foveal thickness (μm) is indicated for each OCT exam. Images correspond to the OCT scans taken 4 weeks after the last (third) injection of each treatment cycle. Baseline image was taken after a 1-month washout period after the prestudy treatment (near-monthly ranibizumab injections). Clear improvement is observed during the two aflibercept treatment cycles, whereas worsening is observed during the two ranibizumab treatment cycles. *CFT* Central Foveal Thickness
- *Visual outcome* BCVA decreased slightly during the first ranibizumab cycle (mean BCVA, -5.1 letters) compared with baseline values. During the first aflibercept cycle, mean BCVA increased with 17.5 letters, the first 15 letters of which were already gained after one aflibercept injection. This 17.5-letter gain was completely lost during the second ranibizumab cycle. However, the mean BCVA increased again with 17.5 letters during the second aflibercept cycle.
- *Safety outcome* Local or systemic adverse events did not occur. In addition, blood pressure and glycaemic values were stable throughout the study period. Glycated haemoglobin values were measured at least once every 3 months and were found to vary between 53.0 and 56.3 mmol/mol, which were similar to prestudy values of 54.1–59.6 mmol/mol.

- *Anatomical outcome* Macular oedema increased slightly during the first ranibizumab cycle (mean CFT, +22 μm) compared with baseline values. In sharp contrast, macular oedema disappeared almost completely during the first aflibercept cycle (Fig. 4). During the first aflibercept cycle, CFT values were reduced by 150 μm (mean decrease in both the eyes). CFT values at the end of the first aflibercept cycle (mean CFT value of 251 μm in both the eyes) were lower than the lowest levels achieved during the previous 2.5 years of ranibizumab treatment. Approximately, half of the anatomical improvement (85 μm) was lost during the second ranibizumab cycle. However, the anatomical status improved again during the second aflibercept cycle (mean CFT value of 228 μm in both the eyes) and exceeded the result obtained during the first aflibercept cycle.

## Discussion

The present double-crossover study compared the efficacy of ranibizumab with that of aflibercept in a patient with bilateral DME. Treatment was prospectively switched three times and we observed very consistent treatment differences favouring aflibercept. The magnitude and consistency of the benefits observed with aflibercept throughout this one-year double-crossover study are striking and warrant clarification. There are 3 potential explanations for the dramatic anatomical and functional success of aflibercept in this setting.

One explanation may be the superior VEGF-binding affinity of aflibercept. Binding affinity of aflibercept to VEGF-A is approximately 100-fold stronger than that of ranibizumab [4]. Theoretically, this leads to a more sustained VEGF inhibition. Still, duration of ranibizumab action in patients with DME, determined by measuring intraocular VEGF suppression, ranges from 27 to 42 days [5]. Because the 4-week interval between each treatment was consistently adhered to, the pharmacokinetic advantage of aflibercept unlikely explains our findings.

A second explanation may be the potential tachyphylaxis or tolerance to ranibizumab. Several studies have suggested the occurrence of tachyphylaxis/tolerance during ranibizumab therapy [6]. Possible mechanisms are cellular (e.g. increased fibrosis), metabolic (e.g. increased expression of VEGF and its receptors) or immunological (e.g. development of neutralizing antibodies). Theoretically, there is a difference between tachyphylaxis and tolerance: tachyphylaxis develops quickly and can be reverted by halting treatment temporarily, whereas tolerance develops slowly and can be partially overcome by increasing dosage or shortening the dosage interval [7]. Before entering the study, the patient was treated with near-monthly ranibizumab injections for 2.5 years. However, we do not believe that tachyphylaxis or tolerance explains the poor response to ranibizumab in the present study because of the following reasons: first, relevant to both tachyphylaxis and tolerance, response to ranibizumab was poor but stable throughout the 2.5-year prestudy treatment as well as during the 1-year study period and second, relevant to tachyphylaxis, despite the 4-month drug holiday between the first and second ranibizumab cycles, the response during the second ranibizumab cycle was superposable to that during the first cycle.

A more plausible explanation might be the different pharmacodynamic properties of the two drugs. Increased levels of VEGF-A [8] in patients with diabetic retinopathy result in VEGF receptor 2-mediated breakdown of the internal blood–retinal barrier (vascular endothelium) leading to DME [9]. In addition to VEGF-A, PlGF-1 (Placental Growth Factor) is also implicated in the pathogenesis of DME [10–12]. PlGF-1 induces VEGF receptor 1-mediated rupture of the external retinal barrier (RPE junctions), thus contributing to diabetic retinal oedema [13]. In addition to its specific and high-affinity binding to VEGF receptor 1, PlGF may indirectly activate VEGF receptor 2 [14], thus disturbing the internal retinal barrier (endothelial cells) along with VEGF-A [15]. Patients with diabetic retinopathy have high vitreous levels of PlGF-1 [16]. Both aflibercept and ranibizumab effectively block vitreous VEGF-A, thereby inhibiting the activation of VEGF receptor 2. In addition, aflibercept, but not ranibizumab, blocks PlGF, thereby inhibiting the binding and activation of VEGF receptors 1 and 2 [4].

Although in exudative AMD the treatment efficacy of ranibizumab and aflibercept seems to be comparable [17], there is possibly a treatment difference between the two drugs in DME. Aetiopathogenesis of macular oedema in diabetes is not identical to that of exudative AMD. Although some aetiopathogenic mechanisms of DME are similar to those of macular oedema in exudative AMD (e.g. increased ocular VEGF activity [9]), other mechanisms are different (e.g. role of PlGF in DME [11]).

We have previously conducted a switch trial with aflibercept in poor responders to ranibizumab in the setting of exudative AMD and noted anatomical and functional benefits after switching patients to aflibercept. Still, in none of the 37 eyes of that study, the extent of the benefit of switching treatment to aflibercept came close to the dramatic aflibercept efficacy we report here (unpublished data from the author).

The major limitation to this study is inherent to the nature of case studies, i.e. results are prone to inter-individual variability of biological systems. Still, its findings are worthwile in giving hints on differential effects of anti-VEGF agents in different retinal diseases and seem in line with those of the DRCR study (protocol T), a large head-to-head study between aflibercept and ranibizumab in patients with DME. This study equally suggests better efficacy of aflibercept compared to ranibizumab, in patients with worse levels of initial visual acuity (less than 69 EDTRS letters) [18]. The DRCR-T study however was conducted mainly in treatment-naïve patients and with a ranibizumab dose of 0.3 mg, a dose unique to the United States.

In conclusion, the results of this 12-month double-crossover case study show that aflibercept can be used to effectively treat DME in eyes with resistance to ranibizumab. Our findings suggest a possible benefit of aflibercept over ranibizumab for treating DME and highlight the role of PlGF and VEGF receptor 1 in the aetiopathogenesis of DME.

## Electronic supplementary material

Supplementary material 1 (DOCX 39 kb)

Supplementary material 2 (TIFF 19612 kb)
