# Supplementary material for: Aflibercept versus ranibizumab for treating persistent diabetic macular oedema
Source: Int Ophthalmol. 2015 May 20;35(4):603–9. doi: 10.1007/s10792-015-0081-7 (PMC4488481; doi:10.1007/s10792-015-0081-7)
Supplement: Supplementary file 1 — Supplementary material 1 (DOCX 39 kb) [file 10792_2015_81_MOESM1_ESM.docx]

| **Table 1.** Patient characteristics at baseline | |
| --- | --- |
| Age, y | 69 |
| Gender | male |
| Hba1c (%) | 7.1 |
| Duration of DME, y | 4 |
| Duration of prestudy ranibizumab injections, mo | 27 |
| Total number of prestudy ranibizumab injections, *n* |  |
| RE* | 23 |
| LE | 21 |
| Baseline ETDRS BCVA |  |
| RE | 60.1 |
| LE | 65.1 |
| Baseline CFT, μm |  |
| RE | 305 |
| LE | 453 |
|  |  |
| Baseline =  *including 2 early injections at an external institute, 4 years before the study  DME = diabetic macular oedema  BCVA = best-corrected visual acuity  ETDRS = Early Treatment of Diabetic Retinopathy Study  CFT = central foveal thickness | |
