# Supplementary figures and images for: Aflibercept versus ranibizumab for treating persistent diabetic macular oedema
Source: Int Ophthalmol. 2015 May 20;35(4):603–9. doi: 10.1007/s10792-015-0081-7 (PMC4488481; doi:10.1007/s10792-015-0081-7)

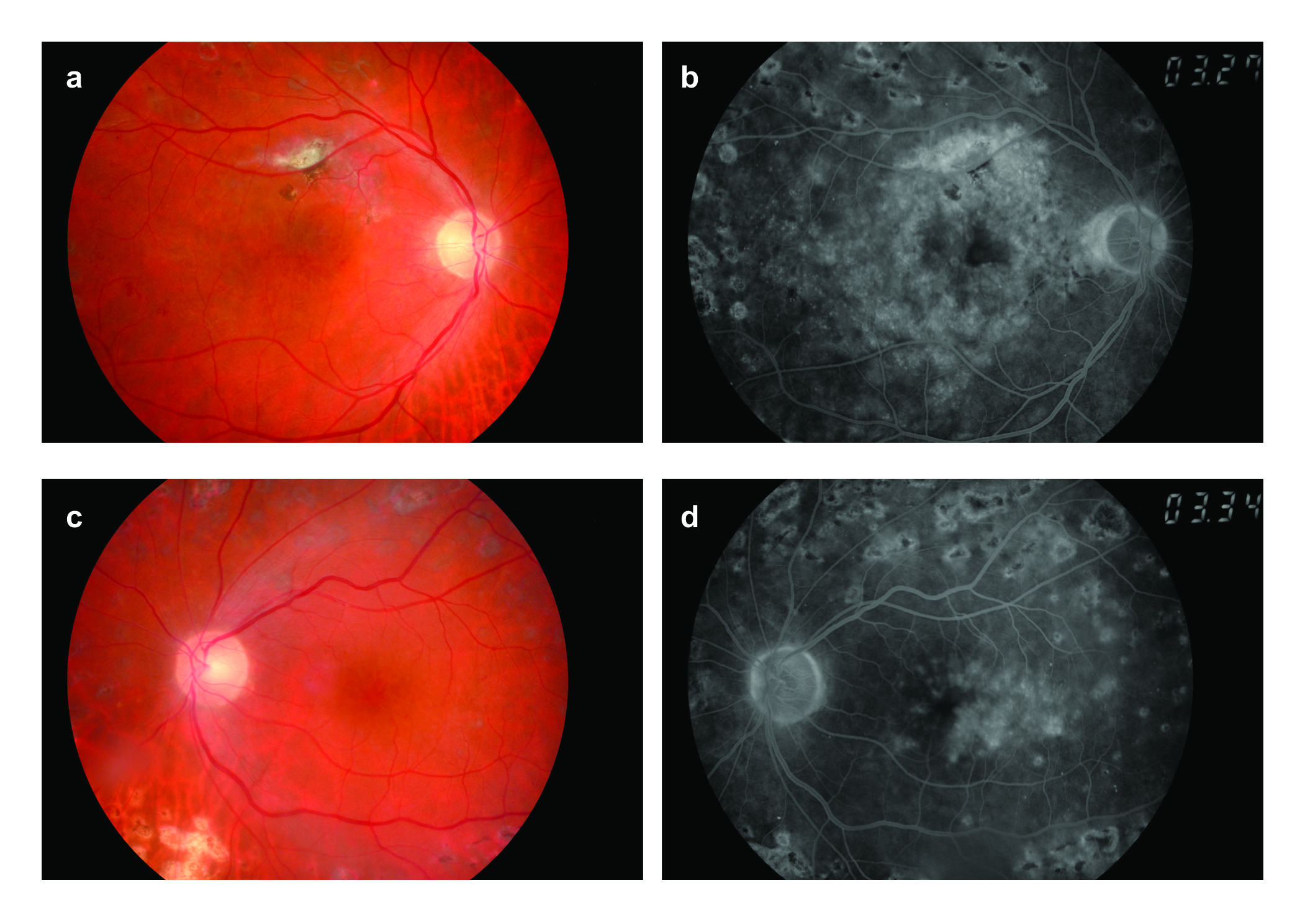

Supplement: Supplementary file 2 — Supplementary material 2 (TIFF 19612 kb) [file 10792_2015_81_MOESM2_ESM.tif]
